# An expanded search for simian foamy viruses (SFV) in Brazilian New World primates identifies novel SFV lineages and host age-related infections

**DOI:** 10.1186/s12977-015-0217-x

**Published:** 2015-11-14

**Authors:** Cláudia P. Muniz, Hongwei Jia, Anupama Shankar, Lian L. Troncoso, Anderson M. Augusto, Elisabete Farias, Alcides Pissinatti, Luiz P. Fedullo, André F. Santos, Marcelo A. Soares, William M. Switzer

**Affiliations:** Departamento de Genética, Universidade Federal do Rio de Janeiro, Rio de Janeiro, Brazil; Laboratory Branch, Division of HIV/AIDS Prevention, Centers for Disease Control and Prevention, 1600 Clifton Rd., MS G45, Atlanta, 30329 USA; Fundação RIOZOO, Rio de Janeiro, Brazil; Centro de Primatologia do Rio de Janeiro, Rio de Janeiro, Brazil; Programa de Genética, Instituto Nacional de Câncer, Rio de Janeiro, Brazil

**Keywords:** Neotropical, Nonhuman primates, Simian foamy virus, Co-evolution, Brazil, Prevalence, Epidemiology, Zoonoses

## Abstract

**Background:**

While simian foamy viruses have co-evolved with their primate hosts for millennia, most scientific studies have focused on understanding infection in Old World primates with little knowledge available on the epidemiology and natural history of SFV infection in New World primates (NWPs). To better understand the geographic and species distribution and evolutionary history of SFV in NWPs we extend our previous studies in Brazil by screening 15 genera consisting of 29 NWP species (140 monkeys total), including five genera (*Brachyteles, Cacajao, Callimico, Mico*, and *Pithecia*) not previously analyzed. Monkey blood specimens were tested using a combination of both serology and PCR to more accurately estimate prevalence and investigate transmission patterns. Sequences were phylogenetically analyzed to infer SFV and host evolutionary histories.

**Results:**

The overall serologic and molecular prevalences were 42.8 and 33.6 %, respectively, with a combined assay prevalence of 55.8 %. Discordant serology and PCR results were observed for 28.5 % of the samples, indicating that both methods are currently necessary for estimating NWP SFV prevalence. SFV prevalence in sexually mature NWPs with a positive result in any of the WB or PCR assays was 51/107 (47.7 %) compared to 20/33 (61 %) for immature animals. Epidemiological analyses revealed an increase in SFV prevalence with age in captive *Cebus* monkeys. Phylogenetic analysis identified novel SFVs in *Cacajao,Leontopithecus,* and *Chiropotes* species that had 6–37 % nucleotide divergence to other NWP SFV. Comparison of host and SFV phylogenies showed an overall cospeciation evolutionary history with rare ancient and contemporaneous host-switching for *Saimiri* and *Leontopithecus* and *Cebus xanthosternos*, respectively.

**Conclusions:**

We identified novel SFV in four neotropical monkey genera in Brazil and demonstrate that SFV prevalence increases with age in *Cebus* monkeys. Importantly, our test results suggest that both molecular and serological screening are currently required to accurately determine infection with NWP SFV. Our study significantly expands knowledge of the epidemiology and natural history of NWP SFVs. The tools and information provided in our study will facilitate further investigation of SFV in NWPs and the potential for zoonotic infection with these viruses.

## Background

Foamy viruses (FV) are complex retroviruses that infect different mammalian orders, including bovine, equine, feline and simian [1]. In addition, humans occupationally and naturally exposed to nonhuman primates (NHPs) via hunting and butchering or keeping NHP pets, or living commensally with NHPs, can be infected with simian FV (SFV) through zoonotic transmissions [2–4]. Recently, FVs have been found in bats [5] and as endogenous elements in sloths [6], aye–aye [7], Cape golden mole [8, 9] and the coelacanth [10] and platyfish [11], with the latter indicating a likely sea origin for these viruses. Most studies with SFV have been conducted with viruses infecting Old World monkeys and apes (OWMAs), which have shown that SFV are widely distributed among diverse species of African and Asian NHP, and have been identified in high prevalence (~70 %) in captive adult primates [1, 12–15]. Less is known about SFV prevalence among wild NHP, but high rates have been observed in some species, such as rhesus macaques (97 %) [16], chimpanzees (44–100 %) [17] and wild colobus monkeys of Côte d’Ivoire (86 %) [18].

South and Central America are home to the parvorder Platyrrhini, also known as New World primates (NWP), which comprise three families, Cebidae, Atelidae, and Pitheciidae, consisting of at least 110 different species of neotropical primates [19]. In the 1970s and 1980s, the presence of SFV infecting NWP was first identified in cell cultures of saliva specimens from spider monkeys (*Ateles sp*.), capuchins (*Cebus sp.*), red-uacaris (*Cacajao rubicundus*) and common marmosets (*Callithrix jacchus*) [12, 20–22]. Thirty-four years later in 2007 the SFVspm lineage infecting a spider monkey was completely sequenced [23], while complete genomes from SFVsqu (squirrel monkey, *Saimiri sciureus*) and SFVmar (common marmoset, *Callithrix jacchus*) were obtained in 2010 [24]. Considering the wide diversity of platyrrhines and the reported coevolution of exogenous SFV with its simian hosts [9, 25, 26], the molecular characterization of only three complete NWP SFV genomes reflects insufficient genetic knowledge of this viral group. We recently PCR-tested a large collection of genomic DNA (n = 332) comprising 14 genera and 41 species of NWP from Brazil and described SFV infection in nine genera (*Alouatta, Aotus, Callithrix, Cebus, Leontopithecus, Saguinus, Saimiri, Callicebus,* and *Chiropotes*) comprising all three Platyrrhini families, including infection in 19 of 65 (29 %) wild howler monkeys (Alouatta species) [26]. In addition, we molecularly characterized two novel SFV lineages infecting the *Cebus* and *Alouatta* genera tentatively named SFVcap and SFVhow, respectively [26]. More recently, one study also demonstrated SFV infection using serology and PCR in a small number of three different NWP species captive in the U.S., including howler, capuchin, and squirrel monkeys [27]. Thus, the geographic distribution and epidemiology of enzootic SFV infection in NWPs is far from complete.

To further improve our understanding of the distribution and prevalence of SFVs in NWPs, we extend our previous studies in Brazil by screening 15 genera consisting of 29 NWP species, including five genera (*Brachyteles, Cacajao, Callimico, Mico*, and *Pithecia*) not previously tested. In addition, monkeys were tested using a combination of both serology and PCR to more accurately estimate SFV prevalence. We also evaluated both screening methods for the detection of SFV in different NWP genera from captive animals living at the Primatology Center of Rio de Janeiro (CPRJ) and the Zoo of Rio de Janeiro (RIOZOO). Finally, in order to better understand the epidemiology of NWP SFV infection, we investigate potential transmission factors for captive animals housed in vivaria.

## Methods

### Study population and sample preparation

Blood samples were collected from 140 NWP housed at the Centro de Primatologia do Rio de Janeiro (CPRJ, n = 78) and the Fundação Jardim Zoológico da Cidade do Rio de Janeiro (RIOZOO, n = 62), consisting of 13 and 8 genera, respectively (Table 1). Whole blood specimens were collected during routine clinical exams by veterinarians at each center with animals under anesthesia. The study was authorized by the governmental animal care and use organization IBAMA (Instituto Brasileiro do Meio Ambiente e dos Recursos Naturais Renováveis, Brazil; permanent license number 11375-1). Plasma was separated from blood cells by centrifugation, collected and stored at −80 °C for serological tests. Peripheral blood mononuclear cells (PBMC) were isolated from whole blood by Ficoll-Paque™ Plus centrifugation. Genomic DNA was extracted from PBMC with the Illustra Blood Genomic Prep Mini Spin kit (GE Healthcare) and stored at −20 °C for future use. DNA quantification was measured by using a NanoPhotometer (Implen).

**Table 1:** Study population and comparison of serological and molecular assay testing

Center	Subfamily	Scientific name	Common name^a^	Total	EIA pos	WB pos	*pol* (192-bp) PCR pos	LTR-*gag* (398-bp) PCR pos	*pol* (520-bp) PCR pos
CPRJ	Alouattinae	*Alouatta guariba*	Southern brown howler monkey	2	1	1	1	0	1
	Aotinae	*Aotus nigriceps*	Black-headed night monkey	1	0	0	0	0	0
		*Aotus* species	Night monkey	1	0	0	0	0	0
	Atelinae	*Ateles paniscus*	Guiana spider monkey	1	0	0	0	0	0
		*Brachyteles arachnoides*	Woolly spider monkey	1	0	0	0	0	0
	Callicebinae	*Callicebus moloch*	Red-bellied titi monkey	1	0	0	0	0	0
		*Callicebus personatus*	Northern masked titi monkey	2	0	0	0	0	0
	Callitrichinae	*Callithrix aurita*	Buffy-tufted-ear marmoset	3	2	2	2	0	0
		*Callithrix geoffroyi*	Geoffroy’s tufted-ear marmoset	1	1	1	1	0	1
		*Callithrix jacchus*	White-tufted-ear marmoset	1	0	0	0	0	0
		*Leontopithecus chrysomelas*	Golden-headed lion tamarin	16	4	2	4	0	1
		*Leontopithecus chrysopygus*	Black lion tamarin	10	0	0	0	0	0
		*Leontopithecus rosalia*	Golden lion tamarin	5	2	2	2	0	1
		*Mico chrysoleucus*	Golden-white tassel-ear marmoset	2	0	0	0	0	0
		*Mico humeralifer*	Black and white tassel-ear marmoset	1	1	1	0	0	0
		*Saguinus bicolor*	Pied bare-faced tamarin	2	0	0	0	0	0
		*Saguinus midas*	Golden-handed tamarin	4	1	1	0	0	0
	Cebinae	*Cebus apella/cay/libidinous*	Brown capuchin	2	1	1	1	0	0
		*Cebus robustus*	Robust tufted capuchin	6	4	3	1	0	3
		*Cebus* species	Capuchin	1	1	1	0	0	0
		*Cebus xanthosternos*	Yellow-breasted capuchin	10	7	7	3	0	1
	Pitheciinae	*Cacajao melanocephalus*	Golden-backed black uakari	3	2	2	1	2	1
		*Pithecia monachus*	Geoffroy’s monk saki	1	0	0	0	0	0
	Saimirinae	*Saimiri sciureus*	Common squirrel monkey	1	0	0	0	0	1
Total (%)				78	27 (34)	24 (31)	16 (20)	2 (3)	10 (13)
RIOZOO	Alouattinae	*Alouatta belzebul*	Red-handed howler monkey	1	1	1	1	0	1
		*Alouatta guariba*	Southern brown howler monkey	3	3	3	3	2	1
		*Alouatta seniculus*	Colombian red howler monkey	2	2	2	2	2	0
	Aotinae	*Aotus* species	Night monkey	11	2	1	1	0	0
	Callicebinae	*Callicebus* species	Titi monkey	3	0	0	0	0	0
	Callitrichinae	*Callimico* *goeldii*	Goeldii’s monkey	1	0	0	1	0	0
	Cebinae	*Cebus apella/cay/libidinous*	Brown capuchin	13	13	13	5	0	0
		*Cebus flavius*	Blonde capuchin	4	3	3	2	0	0
		*Cebus olivaceus*	Weeper capuchin	1	0	0	1	0	0
		*Cebus robustus*	Robust tufted capuchin	4	3	3	0	0	0
		*Cebus species*	Capuchin	1	0	0	0	0	0
		*Cebus xanthosternos*	Yellow-breasted capuchin	9	7	7	7	0	1
	Pitheciinae	*Chiropotes* species	Bearded saki monkey	1	1	1	1	0	1
		*Pithecia* species	Saki monkey	2	0	0	0	0	0
	Saimirinae	*Saimiri* species	Squirrel monkey	1	0	0	0	0	0
		*Saimiri ustus*	Golden-backed squirrel monkey	5	3	3	0	0	1
Total (%)				62	38 (61)	37 (60)	24 (39)	4 (6)	5 (8)
Grand Total (%)				140	65 (46)	61 (43)	40 (29)	6 (4)	15 (11)

*Pos* positive, *WB* western blot, *pol* polymerase, *CPRJ* Centro de Primatologia do Rio de Janeiro, *RIOZOO* Fundação Zoo of Rio de Janeiro

^a^Common names from the International Union for Conservation of Nature and Natural Resources (IUCN) website (http://www.iucnredlist.org/)

### SFV serology

Plasma samples were screened for SFV antibodies by enzyme immunoassay (EIA) and confirmed using a combined antigen Western blot (WB) assay [28]. To broadly detect SFV from NWPs, we used antigens from lysates of Cf2Th cells infected with SFV from a common marmoset (*Callithrix jacchus*, SFVcja ATCC VR-919) or spider monkey (*Ateles* species, SFVasp) isolated in our lab. Using two SFV antigens from highly divergent host species representing two of the three NWP families (Cebidae and Atelidae, respectively) facilitates detection of divergent NWP SFV similar to serological assays we have successfully developed and applied for OWMA SFV testing [28]. Protein concentrations of the lysates were determined using the BioRad DC Protein Assay (Hercules, CA). For the EIA, serum or plasma samples were diluted 1:100 in assay diluent and tested in duplicate in two different microtiter wells coated with crude cell lysates from Cf2Th cells infected with both SFVasp and SFVcja in a single well and uninfected Cf2Th lysates in a separate well to assess assay specificity. Plates were incubated at 37 °C for 1 h. Unbound antibody was removed by washing and a 1:10,000 dilution of peroxidase-conjugated IgG was added and incubated for 30 min at 37 °C. Following another wash step, 100 ul of tetramethlybenzidine (TMB) substrate was added and incubated at 25 °C in the dark for 15 min. Color development was stopped using 1 N H_2_SO_4_. Optical densities (ODs) were measured at 450 nm with a reference at 630 nm. Replicate sample OD values were averaged and adjusted ODs were calculated by subtracting the average ODs of reactivity to the uninfected antigens from those of the combined NWM SFV antigens. An adjusted OD ≥0.235 was set as a cutoff value for seroreactive samples using receiver operator curves (ROC) generated in the MedCalc software program based on assay validation with WB-confirmed specimens described in detail in another study [29].

For WB testing, plasma or serum samples were diluted 1:50 and reacted separately to 150 µg of infected (combined SFVcja and SFVasp antigens) and uninfected cell lysates overnight at 4 °C after protein separation through 4–12 % polyacrylamide gels and transferred to Nytran membranes, as previously described [28]. Seroreactivity was detected using peroxidase-conjugated protein A/G (Pierce, Rockford, IL) and chemiluminescence (Amersham, Uppsala, Sweden) [28]. Samples with seroreactivity to both Gag p68 and p72 precursor proteins with an absence of similar reactivity to antigen from uninfected Cf2Th cells was interpreted as seropositive. Specimens without reactivity to either Gag protein were considered seronegative. Animals testing EIA negative but PCR-positive were also tested using the WB assay to confirm the serological screening results.

Both assays were validated in another study using plasma/serum from 104 PCR-positive or PCR-negative NWPs and both were shown to have sensitivities and specificities >94 % [29].

### Molecular tests

To confirm the integrity of the extracted genomic DNA (gDNA) and to verify the primate host species taxonomic classification, we PCR-amplified a 975-bp cytochrome B (*cytB*) mitochondrial (mtDNA) sequence from each monkey in our study using 100 ng gDNA as described previously [26]. All samples positive in this assay were considered suitable for SFV detection using PCR testing. Phylogenetic analysis of selected *cytB* sequences was done to confirm NWP species as described below.

To detect SFV proviral DNA in NWP, we first performed a screening PCR for short polymerase (*pol*) sequences using generic primers and 100 ng gDNA as previously described [26]. The outer primers SIF5 N 5′-tacatggttataccccackaaggctcctcc-3′ and SIRN 5′- aataawggataccactttgtaggtcttcc-3′ and semi-nested primers SIP4n 5′-gcattccgatcaaggatcagcatt-3′ and SIRN, were used to generate a 192-bp fragment using standard PCR conditions [26]. This nested PCR assay has a high sensitivity for detection of diverse NWP SFV variants with a reported 97.2 % diagnostic accuracy, 100 % sensitivity, and 91 % specificity when compared with WB results for 47 seronegative and 59 seropositive NWPs [26]. In addition, using cloned *pol* sequences from SFVasp and SFVcja we have shown that the assay can detect between 1–10 copies each, which is similar to that reported recently using an SFVsqu *pol* plasmid [27, 29]. Generic primers were also used with 500 ng NWP DNA to amplify and sequence two additional SFV genomic regions, including a 398-bp LTR/*gag*-sequence (225-bp in LTR and 173-bp in *gag*) and a 520-bp *pol* fragment using nested PCR [26]. Infection status using PCR testing is defined as PCR positivity in any of the three PCR assays used in our study. For the diagnostic *pol* PCR assay, each sample was tested in triplicate in three different assay runs and a sample was considered positive if any of the replicates tested positive. Amplified products were sequenced on both strands with the Big Dye v.3.1 kit (Life Technologies, Carlsbad, USA) using an automated ABI 3130XL Genetic Analyzer.

SFV and *cytB* sequences were edited with SeqMan v7.0 (DNASTAR, Madison, USA) and were aligned separately using Clustal W implemented in either BioEdit or MEGA6 [30] with those respective sequences available from NWP at GenBank. The best fitting distance model of nucleotide substitution for each alignment was inferred using the ML method with goodness of fit measured by the Bayesian information criterion in MEGA6. SFV and host sequences from a chimpanzee and African green monkey were used as outgroups in the respective phylogenetic analyses. The best fitting nucleotide substitution model for the SFV phylogenetic alignments was inferred to be the Tamura 3-parameter model (T3P) with discrete gamma (Γ) rate variation and was used for the ML analysis with 1000 bootstrap replicates to assess strength of the inferred relationships. Nucleotide identities were determined using Geneious v8.1.5.

Phylogenetic relationships and time to most recent common ancestors (TMRCA) of the SFV *pol* sequences were co-inferred using Bayesian analysis with the BEAST v1.8.1 program [35] and the Hasegawa-Kishino-Yano (HKY) model with discrete gamma (Γ) rate variation (0.5) inferred from the alignment using MEGA6. Statistical support for the inferred Bayesian trees was assessed by posterior probabilities. For the Bayesian phylogenetic analyses, an uncorrelated lognormal relaxed molecular clock model was used and each run consisted of two independent 100 million Markov chain Monte Carlo (MCMC) generations with sampling every 10,000th generation and a constant coalescent tree prior. The relaxed clock was calibrated at two nodes using normal distributions for the Cercopithecidae-Hominidae split 29 million years ago (MYA) with a standard deviation (SD) of 6 and the Catarrhini and Platyrrhini split 43 MYA with an SD of 4.5. These calibration points are based on fossil records as described in Perelman et al. [31]. Convergence of the MCMC was assessed by calculating the effective sampling size (ESS) of the runs using the program Tracer v1.6.0 (http://www.beast.bio.ed.ac.uk/Tracer). All parameter estimates showed significant ESSs >300, indicating sufficient mixing. The tree with the maximum product of the posterior clade probabilities (maximum clade credibility tree) was chosen from the posterior distribution of 9001 sampled trees after burning in the first 1000 sampled trees with the program TreeAnnotator version 1.8.1 [36]. Two tree prior speciation models, the Yule process and birth–death process, were compared using marginal likelihood estimation (MLE) using path sampling and stepping-stone sampling implemented in Beast 1.8.1, to infer the best tree prior for the data.

The 42 new *cytB* sequences from this study were aligned with 164 reference sequences from a broad representation of NWM species available at GenBank, including 13 from our previous study [25]. The best fitting model for the *cytB* alignment inferred was the general time reversible (GTR) model with discrete gamma rate variation (Γ, 1.20) and a proportion of invariable sites (I, 0.37) and was used for ML analysis with 1000 bootstrap replicates.

All new *cytB* and SFV sequences generated during our study have been deposited at GenBank with the accession numbers KR528388–KR528428 and KR528429–KR528448, respectively.

### Statistical analyses

To better understand the epidemiology of SFV in NWPs, differences in SFV prevalence between monkeys born in captivity and in the wild, between juvenile and adult monkeys, and between males and females were evaluated with Fisher’s exact tests and two-tailed *p*-values. Differences in ages of SFV-infected and uninfected animals were assessed using Student’s *t* tests. Categorization of juveniles for distinct genera was assigned according to a cut-off of 0.67 of the age to be considered as adult (which varied from 1.2–1.5 year for *Callithrix* to 4–8 year for *Cebus*). *Cebus* monkeys, the most common genus [36 % of the study population (Table 1)], were defined as adults with a cut-off age of 4 years for male and 8 years for female [32], and comparisons of SFV prevalence was also evaluated independently in this genus. SFV prevalence was evaluated in individual vivaria harboring primate familial groups and also verified in alpha couples. Comparison of differences between prevalence determined using the screening assays was done using two-tailed z-ratio tests in GraphPad.

## Results

### Study population

Of the 140 animals, 64 were male and 76 were female. While there was about equal numbers of wild- and captive-born animals at both institutions, there were more wild-born animals at the RIOZOO (n = 42) compared to CPRJ (n = 25). Since we did not have ages for 23/140 (16 %) monkeys, the majority of which were at the RIOZOO (n = 19) or were wild-born (n = 16), we estimated their ages based on sexual maturity and combined this information with the sexual maturity of animals with known birth dates. More NWPs were mature (n = 107) compared to immature (n = 33) and there were nearly equal amounts of mature and immature animals at each institution. For those 117 NWPS with known birth dates, ages ranged from 1.3 to 21.6 years with a mean and media of 7.0 and 6.5 years, respectively, supporting our estimate that most animals in the study were mature. A total of 67 animals were housed in 36 shared vivaria (22 at CPRJ and 14 at RIOZOO) which consisted of 28 males and 39 females, 45 adults and 22 juveniles, and 39 wild- and 28-captive-born monkeys.

**Fig. 1:**
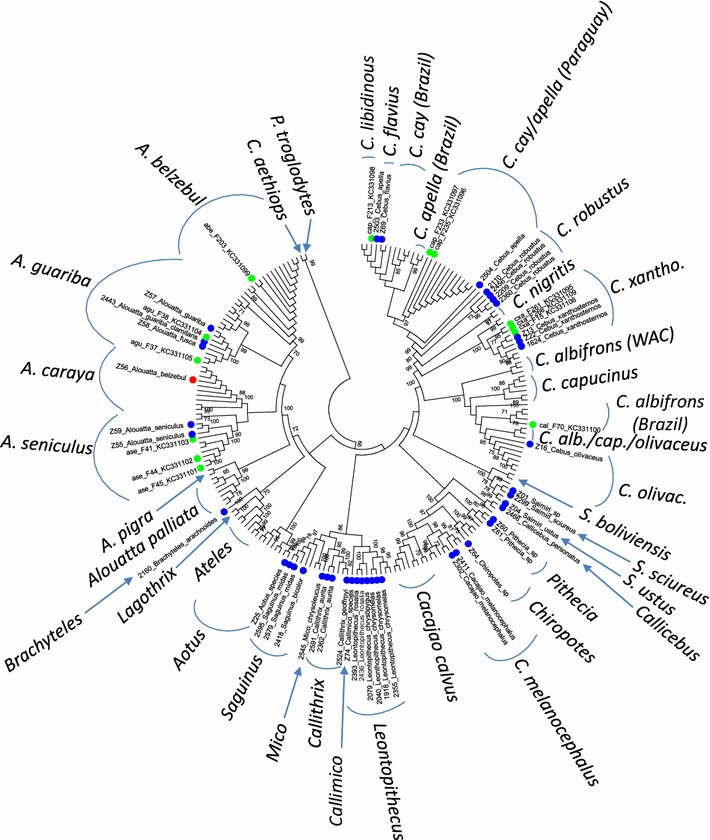
Host phylogeny and taxonomy inferred using maximum likelihood analysis of Platyrrhini mitochondrial cytochrome B sequences. An alignment of 627 nucleotides from 205 taxa was used in the analysis. *Green* and *blue* *circles* represent sequences generated in our first and current studies, respectively; *red circles* indicate new sequences with discordant morphological and phylogenetic taxonomic classification. All other taxa are reference sequences from GenBank. Bootstrap support was determined using 1000 resampling replicates and values ≥70 % are provided at nodes. *WAC* western Amazonian countries (Venezuela, Ecuador, Peru, Bolivia), *C. xantho.* *C. xanthosternos*, *C. olivac.* *C. olivaceus*, *C. alb./cap./olivaceus* mixture of *C. albifrons*, *C. capucinus* *and* *C. olivaceus*. *ase* *Alouatta seniculus*, *agu* *Alouatta guariba*, *abe* *Alouatta belzebul*, *cxa* *Cebus xanthosternos*, *cap* *Cebus apella*, *cal* *Cebus albifrons*

**Fig. 2:**
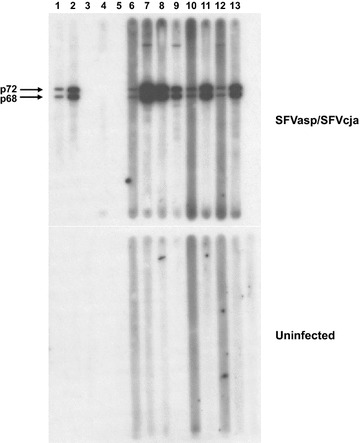
Broad detection of simian foamy virus (SFV) antibodies in neotropical primates from Brazil. *Upper panel* shows seroreactivity of representative neotropical primate plasma samples to the combined NWM SFV antigens from spider monkey (*asp* *Atele*s species) and marmoset (*cja Callithrix jacchus*) cell cultures. *Lower panel* shows reactivity to crude cell lysate antigens from uninfected canine thymocytes (Cf2Th). Seroreactivity was defined for those specimens with reactivity specific to the diagnostic Gag doublet proteins (p68/p72) in the combined viral antigens. Lanes 1 and 2 are from SFV-infected spider and capuchin monkeys, respectively; lanes 3 and 5 are blanks, lane 4 is an uninfected human control; lanes 6–13 are from *Saimiri ustus, Alouatta guariba, Cacajao melanocephalus, Chiropotes* species*, Leontopithecus chrysomelas, Alouatta seniculus, Saimiri sciureus* and *Leontopithecus rosalia*, respectively

To confirm both the genomic DNA integrity and morphology-based taxonomic classification performed by biologists and veterinarians at the two centers housing the primates, we phylogenetically analyzed mtDNA *cytB* sequences. We were successful in obtaining strong *cytB* PCR signals for all 140 NWPs in this study, indicating that the quality of the DNA extracted from all 140 blood samples was suitable for PCR testing. In addition, we observed very high congruence between the morphological and phylogenetic classifications for all monkeys selected for *cytB* phylogenetic analysis (Fig. 1) with the exceptions described below. The majority of monkeys at CPRJ were in the Callitrichinae subfamily, while most zoo monkeys were in the Cebinae subfamily (Table 1). Two Brazilian monkeys (F233, F235) identified as *C. apella* from a previous study [26] clustered with *C. cay* from Paraguay with good support (bootstrap = 88) but not with *C. apella* reference sequences, suggesting they are *C. cay* and not *C. apella* (Fig. 2). However, classification of *C. cay* as a separate species is not fully supported yet as deeper studies in other geographical areas are required to confirm its species status. Thus, herein we will combine *C. cay*, *C. libidinous* and *C. apella* as a single group for analysis. Similarly, capuchin monkeys have recently been suggested to be taxonomically divided into two genera, *Sapajus* for capuchins without head tufts, also known as robust capuchins, which comprises most previous *Cebus* species, and the remaining *Cebus* consisting of *C. olivaceus, C. albifrons and C. capucinus,* also known as gracile capuchins [33]. Indeed, our phylogenetic analysis strongly support this classification of capuchins into two genera (Fig. 1); however, for consistency with the nomenclature used in our previous report we prefer to use *Cebus* herein [26]. Phylogenetic determinations of the cytB species for monkey Z56, morphologically identified as an *Alouatta belzebul*, indicated it is an *A. caraya* by strongly clustering (bootstrap = 94) within the *A. caraya* clade (Fig. 2), but interestingly is a species not present at that zoo and which was also not present in our study population. These results were confirmed with repeat sequencing and phylogenetic analysis (data not shown).

### SFV seroprevalence

All 140 primate plasma samples were tested for the presence of SFV antibodies with EIA and WB methods. By EIA screening, 65/140 (46.4 %) samples were found positive (Table 1). Twenty-seven were from CPRJ, corresponding to 34.6 % (27/78) of primates tested at that center, while at RIOZOO EIA seropositivity was 61 % (38/62). EIA-positive samples were further subjected to WB analysis to confirm the presence of SFV antibodies. Of the 65 EIA-positive samples, 61 (95.3 %) were WB-positive, of which 24 were from CPRJ (31 %; 24/78) and 37 were from RIOZOO (60 %; 37/62) (Fig. 1). Seropositivity was observed amongst all NWP subfamilies included in the study except for Atelinae and Callicebinae but which included only nine samples total (6.4 %). Four monkeys were EIA-positive but WB-negative, including one *Aotus* species (Z28; male, 2.1-years-old), one *Cebus robustus* (2207; male, 8.1-years-old) and two *Leontopithecus chrysomelas* (1029 and 1859; female and male, 21.6 and 12.8-years-old, respectively). Adjusted ODs for these four samples were near the EIA cutoff and WB testing was negative at least twice suggesting these EIA results are likely due to nonspecific reactivity. A complete list of the infected species and the SFV seroprevalence at each center is shown in Table 1.

### SFV molecular prevalence

We first performed a screening PCR assay [26] to detect 192-bp SFV *pol* sequences in all 140 NWPs. Sixteen of seventy-eight (20.5 %) NWPs at CPRJ were PCR-positive, including six Leontopithecus, five *Cebus*, three *Callithrix*, and one *Alouatta,* all of the Cebidae family, and one *Cacajao* of the Pitheciidae family (Table 1). Of 16 PCR-positive monkeys, five (31.2 %) were SFV seronegative, including one each *Callithrix* aurita (2362), *Cebus cay* (2503), *Cebus robustus* (2456), and two *Leontopithecus chrysomelas* (2040 and 2355). All five animals were captive-born, four are female of which three that were not Cebus were sexually mature, and longer *pol* and LTR-*gag* sequences were not detected in their PBMC DNAs. Similarly, of the 62 monkeys at RIOZOO, 24 (38.7 %) were positive in the screening PCR test, including 13 Cebus, 6 *Alouatta*, 1 *Aotus*, and 1 Callimico (Table 1). Of the 24 PCR-positive zoo monkey specimens, five (20.8 %) monkeys of different species (*Aotus* sp. (Z25), *Cebus xanthosternos* (Z22), *Cebus olivaceus* (Z16), *Cebus flavius* (Z69), and *Callimico* sp. (Z74)) were all EIA and WB negative and PCR negative for longer *pol* and LTR-*gag* sequences. Three of these diagnostic PCR-positive only animals were captive born and female, and two males and one of the females were sexually mature. All ten animals tested positive in the diagnostic PCR assay at least once with a mean and median of 1.7 positive tests, possibly indicating low viral loads in the majority of these monkeys.

### Comparison of serology and molecular screening assays for SFV detection

Given the discordant serological and screening PCR results for some animals, we examined in further detail the serological and the molecular test results of all 140 animals to determine the better screening tool (Table 1). We observed a higher prevalence of SFV-positive monkeys by WB than by the screening PCR test (43 vs 29 %, respectively; p = 0.0045). Of the 140 primates analyzed, 51 % (n = 72) were identified as SFV-infected by at least one method. However, only 30 (21 %) primates from a variety of species were positive using both assays, including seven *Alouatta*, 15 Cebus, four *Leontopithecus,* two *Callithrix,* and one each of *Cacajao* and *Chiropotes* species. Thirty (21 %) monkeys were positive only in the WB assay, including 21 Cebus, two *Saimiri,* and one each of *Aotus*, *Cacajao*, *Callithrix*, *Mico,* and *Saguinus* species. Seventeen (56 %) of these 30 monkeys with discordant results were from the RIOZOO, 18 (60 %) were female, 12 (40 %) were captive-born, and 24 (80 %) were sexually mature. In contrast, 10 (7 %) monkeys were positive only in the screening PCR assay, including five Cebus, two *Leontopithecus,* and one each of *Aotus*, *Callimico*, and *Callithrix* species (Table 1). Half of these 10 monkeys were from RIOZOO and 8 (80 %) were captive-born; six (60 %) were sexually mature and three (30 %) were male.

All 140 monkey DNA samples were subjected to additional PCR testing to confirm the serological and screening PCR tests and to obtain longer SFV *pol* fragments containing adequate sequence information for resolution by phylogenetic analysis. In addition, we tested all 140 monkeys for LTR-gag sequences as we have previously shown the utility of this assay for detecting infection with divergent NWP SFVs [26]. This additional testing identified SFV infection in 14 monkeys using the longer *pol* PCR test and in six monkeys using the LTR-*gag* assay. Seven of the 14 animals positive for the longer *pol* sequences tested negative using the shorter *pol* assay. Five of these seven samples were also WB-positive. Six monkeys positive for LTR-*gag* sequences tested negative in the screening PCR test and longer *pol* PCR assays. However, all six LTR-*gag* positive monkeys were also seropositive. Overall, the LTR-*gag* and longer *pol* PCR assays detected seven additional SFV infections, including two squirrel monkeys that were repeatedly seronegative upon duplicate EIA testing and by WB testing. Therefore, in some instances the PCR assays to specific genomic fragments detected viral sequences when the more generic screening PCR did not, likely reflecting the influence of SFV genome-specific regions on molecular detection. Overall, the SFV prevalence found in our study population using molecular assays was 32.1 % (45/140) compared to 42.8 % that were WB-positive (*p* = 0.0428, Table 1). In all testing, both the positive and negative controls performed as expected supporting the absence of cross-contamination as a source of the discrepant results, though this does not absolutely exclude this possibility.

We next compared the differences in demographic factors for each possible serologic and PCR test combination to evaluate their potential impact on the test outcome (Table 2). We found no significant difference between male vs female, mature vs immature, wild-born vs captive-born, or institute (CPRJ vs RIOZOO) for animals that were WB+/PCR+, WB+/PCR−, WB−/PCR+, WB−/PCR−, respectively, with all *p*-values ≥0.146. For example, while a slightly greater proportion of immature animals were SFV-positive by any assay than sexually mature animals, these results were not significant (*p* = 0.19). To assess whether the efficiency of molecular and serological methods correlated with specific NWP genera, we compared the results of both tests in a genus-specific manner (Table 3). For example, 43 *Cebus* individuals were SFV-positive by serology and/or PCR testing, 19 of which were SFV-positive by both methods, showing 43 % concordance. Nonetheless, 19 (43 %) and five (11.6 %) *Cebus* specimens tested either WB or PCR positive only, respectively. One hundred percent assay concordance was seen for genera *Alouatta* (7/7), *Cacajao* (2/2), *and Chiropotes* (1/1), although the total number of representatives for each genus was low. Assay concordance was not observed for *Aotus* (0 %, 0/2), *Callithrix (*50 %, 2/4), *Leontopithecus* (67 %, 4/6), and *Saimiri* (0 %, 0/4). However, the single *Callimico* specimen was positive using only the screening PCR test, while 1/3 (33 %) *Mico* and 1/6 (17 %) *Saguinus* monkeys were only positive by WB testing. These three genera all belong to the Cebidae family. Though small numbers of animals were tested, SFV was not detected in four genera using either method, including *Ateles*, *Brachyteles*, *Callicebus*, and *Pithecia* (Table 3). Overall, both methods are able to detect SFV in representatives of all three NWP families with a combined estimated SFV prevalence of 51.4 % (Table 3).

**Table 2:** Comparison of simian foamy virus prevalence in New World primates in Brazil defined by Western blot (WB) and PCR testing^a^

Center	N	WB^+^/PCR^+^	WB^+^/PCR^−^	WB^−^/PCR^+^	WB^−^/PCR^−^
CPRJ^b^	78	16 (21 %)	8 (10 %)	6 (8 %)	48 (61 %)
Male	34	6 (18 %)	2 (6 %)	2 (6 %)	24 (70 %)
Female	44	10 (23 %)	6 (14 %)	4 (9 %)	24 (54 %)
Mature	60	9 (15 %)	5 (8 %)	4 (7 %)	42 (70 %)
Immature	18	7 (39 %)	3 (17 %)	2 (11 %)	6 (33 %)
Wild^c^	25	7 (28 %)	2 (8 %)	0	16 (64 %)
Captive^d^	53	9 (17 %)	6 (11 %)	6 (11 %)	32 (61 %)
RIOZOO^e^	62	19 (31 %)	17 (27 %)	6 (10 %)	20 (32 %)
Male	30	10 (33 %)	9 (30 %)	3 (10 %)	8 (27 %)
Female	32	9 (28 %)	8 (25 %)	3 (9 %)	12 (38 %)
Mature	47	14 (30 %)	16 (34 %)	4 (9 %)	13 (28 %)
Immature	15	5 (33 %)	1 (7 %)	2 (14 %)	7 (46 %)
Wild	42	14 (33 %)	13 (31 %)	3 (7 %)	12 (29 %)
Captive	20	5 (25 %)	4 (20 %)	3 (15 %)	8 (40 %)
Total	140	35 (25 %)	25 (18 %)	12 (9 %)	68 (48 %)

^a^Positive for at least one PCR assay (screening polymerase (*pol*), LTR-*gag* and/or longer *pol* sequences)

^b^Primatology Center of Rio de Janeiro

^c^Wild born

^d^Captive born

^e^Zoo of Rio de Janeiro

**Table 3:** Comparison of SFV^a^ prevalence in NWP^b^ genera from Brazil using Western blot (WB) and PCR assays

Family	Genus	n	WB+/PCR+^c^	WB+/PCR−	WB−/PCR+	WB−/PCR−	Overall SFV prevalence (%)
*Atelidae*	*Alouatta*	8	7	0	0	1	7/8 (88)
	*Ateles*	1	0	0	0	1	0/1
	*Brachyteles*	1	0	0	0	1	0/1
*Cebidae*	*Aotus*	13	0	1	1	11	2/13 (15)
	*Callicebus*	6	0	0	0	6	0/6
	*Callimico*	1	0	0	1	0	1/1 (100)
	*Callithrix*	5	2	1	1	1	4/5 (80)
	*Cebus*	51	19	19	5	8	43/51 (84)
	*Leontopithecus*	31	4	0	2	25	6/31 (19)
	*Mico*	3	0	1	0	2	1/3 (33)
	*Saguinus*	6	0	1	0	5	1/6 (17)
	*Saimiri*	7	0	2	2	3	4/7 (57)
*Pitheciidae*	*Cacajao*	3	2	0	0	1	2/3 (67)
	*Chiropotes*	1	1	0	0	0	1/1 (100)
	*Pithecia*	3	0	0	0	3	0/3
	Total (%)	140	35 (25)	25 (17.9)	12 (8.6)	68 (48.6)	72 (51.4)

^a^Simian foamy virus

^b^New World primates

^c^Positive for at least one PCR assay (screening polymerase (*pol*), LTR-*gag* and/or longer *pol* sequences)

### Evolutionary history of SFV in NWPs

**Fig. 3:**
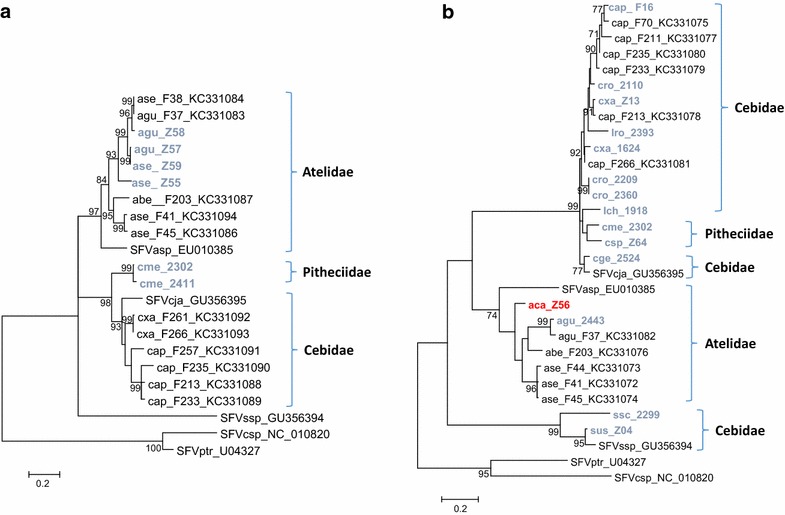
Platyrrhini simian foamy virus (SFV) **a** LTR-*gag* and **b** polymerase phylogenies inferred using maximum likelihood analysis. An alignment of 399 LTR-gag and 343 nucleotides from 22 to 31 taxa, respectively, were used in the analysis. New sequences generated in the current study are in *blue* and *red text*. *Red text* indicates that the phenotypic and genotypic results did not match and genotypic results are provided. Bootstrap support was determined using 1000 nonparametric resampling replicates and values ≥70 % are provided at nodes. *aca* *Alouatta caraya*, *ase* *Alouatta seniculus*, *agu* *Alouatta guariba*, *abe* *Alouatta belzebul*, *asp* *Ateles species*, *cme* *Cajacao melanocephalus*, *cja* *Callithrix jacchus,* *cxa* *Cebus xanthosternos,* *cap* *Cebus apella*, *cro* *Cebus robustus,* *lro* *Leontopithecus rosalia,* *lch* *Leontopithecus chrysomelas, csp Chiropotes* species, *cge* *Callithrix geoffroyi*, *ssc* *Saimiri sciureus,* *sus* *Saimiri ustus*, *ssp* *Saimiri* species, *csp* *Chlorocebus* species, *ptr* *Pan troglodytes*. *Scale bar* is number of substitutions/site

**Fig. 4:**
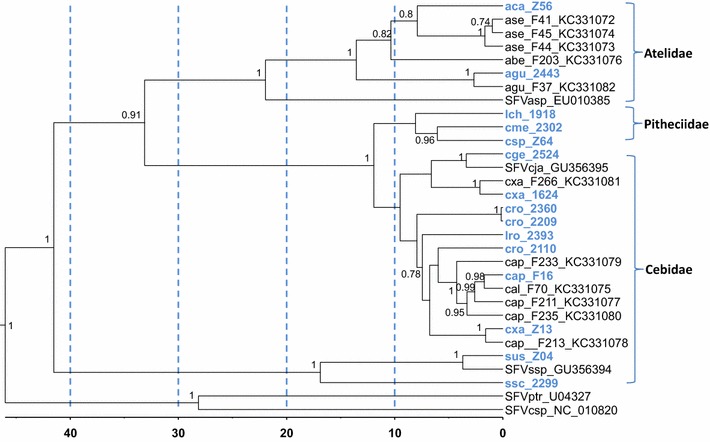
Relative divergence time estimates for simian foamy virus (SFV) polymerase sequences inferred using Bayesian phylogenetic analysis. An alignment of 341 nucleotides from 31 taxa was used in the analysis. New sequences generated in the current study are in *blue text*. Posterior probabilities ≥0.7 are provided at nodes. Scale for divergence date estimates is in 1 million years with *blue dashed lines* every 10 million years. *aca* *Alouatta caraya*, *ase* *Alouatta seniculus*, *agu* *Alouatta guariba*, *abe* *Alouatta belzebul*, *asp* *Ateles* species, *cme* *Cajacao melanocephalus*, *cja* *Callithrix jacchus*, *cxa* *Cebus xanthosternos*, *cap* *Cebus apella*, *cro* *Cebus robustus*, *lro* *Leontopithecus rosalia*, *lch* *Leontopithecus chrysomelas, csp Chiropotes* species, *cge*, *Callithrix geoffroyi*, *ssc* *Saimiri sciureus*, *sus* *Saimiri ustus*, *ssp* *Saimiri* species, *csp* *Chlorocebus* species, *ptr* *Pan troglodytes*

**Fig. 5:**
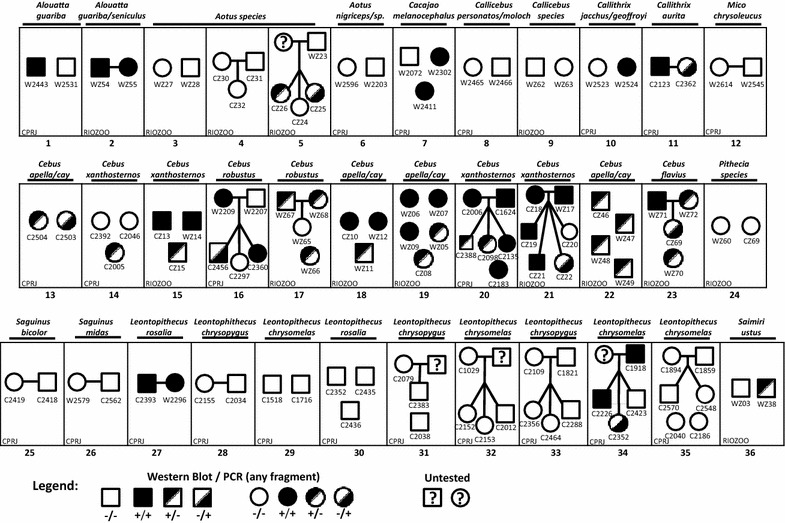
Investigation of simian foamy virus (SFV) transmission in shared vivaria of selected New World primates housed at the Centro de Primatologia do Rio de Janeiro (CPRJ) or the Fundação Jardim Zoológico da Cidade do Rio de Janeiro (RIOZOO). Shared vivaria are represented by boxes. *Numbers below boxes* indicate vivaria or cage number, while primate species is provided above boxes. Legend shows gender (squares, male; circles, female) and SFV infection status as determined by Western blot and/or PCR status using any of the three PCR tests targeting polymerase (200-bp or 500-bp) and LTR-*gag* regions (380-bp). Animal codes are given below gender symbols and “W” or “C” before the code name indicates born in the wild or in captivity, respectively. Institution abbreviation is given in the *vivaria boxes*. *Question marks in symbols* indicate that specimens were not available for testing. *Horizontal lines* indicate sexual pairs and *vertical lines* indicate offspring of mates

In total, six LTR-*gag* and 15 longer *pol* sequences were successfully obtained from the 140 monkeys in our study. Phylogenetic trees were constructed for both genomic regions with these 21 new sequences and with those available at GenBank (Figs. 3, 4, 5). In all phylogenetic reconstructions, we observed a clear divergence of New and Old World primate SFVs, reflecting a co-evolution of SFVs with their simian hosts, as previously suggested [25, 26]. In the LTR-*gag* tree (Fig. 3), a family-specific SFV structure was inferred. The Atelidae clade comprised SFV from *Ateles* and *Alouatta*, respectively. The Pitheciidae clade contained two sequences from *Cacajao melanocephalus* as a sister clade to the Cebidae clade which comprised sequences from *Cebus* and *Callithrix* (SFVcja). The single sequence from a squirrel monkey (SFVssp, *Saimiri* species) formed a long, independent lineage that appears to share a common ancestor with all other NWP LTR-*gag* sequences (Fig. 3a).

In the inferred *pol* ML tree, the family-specific structure is largely conserved, but with some exceptions. For example, the *pol* sequences from a *Cacajao melanocephalus* (2302) and *Chiropotes* species (Z64), both members of the Pitheciidae family, clustered within the Cebidae clade containing sequences from *Cebus* and *Callithrix* (Fig. 3b). Nonetheless, the *Cacajao* and *Chiropotespol* sequences are 8–35 % divergent from other NWP SFV. The *Leontopithecus chrysomelas* sequence (Lch_1918) clustered weakly with the Pitheciidae SFV, whereas the *L. rosaliapol* sequence (Lro_2393) clustered with Cebus SFV instead of both *Leontopithecu*s sequences forming a genus-specific clade. The *Leontopithecuspol* sequences are 10–37 % divergent from other NWP SFV. As in the LTR-*gag* tree, the *Saimiri* sequences formed a lineage independent of other Cebidae and contained the new *Saimiri sciureus* (Ssc_2299) and *S. ustus* (Sus_Z04) *pol* sequences with Sus_Z04 being closer phylogenetically to SFVssc from a captive squirrel monkey identified as *S. sciureus* (Fig. 3b) [24]. The sequence from zoo animal Z56 identified as *Alouatta caraya* by *cytB* phylogenetic analysis clustered basally to all other *Alouatta* SFV from three different species with species-specific lineages, likely supporting further Z56 as an *A. caraya*.

The ML results were nearly mirrored in the Bayesian inferred tree (Fig. 4) with strong statistical support except for the three Pitheciidae sequences from Lch_1918, Cme_2302 and Csp_Z64 that formed a sister clade basal to the Cebidae SFV from *Cebus* and *Callithrix* with high posterior probability (PP = 1), instead of clustering between the *Cebus* and *Callithrix* SFV as in the ML-inferred topology. Two Bayesian trees were inferred using both the birth–death and the Yule speciation process models and which gave identical topologies (data not shown). However, the birth–death model was preferred by MLE analysis and was also used to infer SFV divergence times.

TMRCAs for Haplorhini, Catarrhini, and Platyrrhini SFVs were estimated to be 46.44 MYA (95 % high posterior density intervals (HPD) 35.63–58.38 MYA), 28.07 MYA (95 % HPD 18.73–37.36 MYA), and 41.63 MYA (95 % HPD 33.25–50.22 MYA), respectively (Table 4, Fig. 4). While the inferred TMRCAs for Haplorhini and Catarrhini SFVs are strongly consistent with previous estimates using SFV *pol* sequences [26], and those inferred from both simian genomic sequences and from fossil estimates [31], TMRCA for Platyrrhini are about 10 MY older but with 95 % HPDs that overlap previous TMRCA estimates for Platyrrhini *cytB* evolution [26]. Although fossil estimates are not available for Atelidae, Alouattinae, Cebinae, Saimirinae, Callitrichinae, and Pitheciinae, the divergence times for the majority of SFV in these families have 95 % HPDs that overlap with those from previous host studies, supporting our SFV TMRCA estimates [26, 31, 34]. The sole exception is the Saimirinae node, which was inferred to have a higher TMRCA in the current study then that expected under the co-evolution hypothesis, likely by the addition of the new, divergent *pol* sequence from *S. sciureus* (2299) to the analysis, but for which TMRCA was not determined in the Perelman et al. study [31].

**Table 4:** Time to most recent common ancestor (TMRCA) mean estimates for Haplorhini and simian foamy virus (SFV) polymerase (*pol)* and simian host sequences in million years ago

Branch node	TMRCA SFV *pol* ^*a*^	TMRCA SFV *pol* ^b^	TMRCA *cytB* ^*b*^	TMRCA simian phylogeny^c^	Fossil estimate^c^
Haplorhini	46.44 (35.63–58.38)	42.38 (33.86–51.11)	42.37 (34.26–51.27)	43.47 (38.55–48.36)	43 ± 4.5
Catarrhini	28.07 (18.73–37.36)	24.33 (15.52–35.17)	24.17 (15.1–35.3)	31.56 (25.66–37.88)	29 ± 6.0
Platyrrhini	41.63 (33.25–50.22)	28.11 (15.02–45.21)	34.58 (20.43–49.7)	24.82 (20.55–29.25)	23.5 ± 3.0
Atelidae	21.94 (14.00–31.94)	15.55 (6.12–31.21)	20.55 (8.21–37.14)	16.13 (10.52–21.35)	NA
Atelinae	ND	3.4 (0.75–9.27)	ND	11.25 (7.25–15.46)	NA
Alouattinae	13.55 (8.14–20.79)	9.06 (3.64–18.47)	7.89 (2.4–17.85)	6.03 (3.74–8.57)	NA
Cebinae	7.94 (5.17–14.05)	3.89 (1.32–8.57)	13.1 (5.96–23.24)	6.00 (3.13–9.35)	NA
Saimirinae	16.87 (8.55–27.55)	3.37 (0.75–9.27)	5.4 (1.48–12.62)	ND	NA
Callitrichinae	3.37 (1.13–6.74)	3.21 (0.62–7.8)	2.79 (0.4–8.67)	8.42 (5.72–11.38)	NA
*Cacajao/Chiropotes* split	6.05 (2.31–11.03)	ND	ND	7.51 (4.36–10.88)	NA

*NA* not available, *ND* not determined

^a^Using a 341-bp alignment for 31 SFV taxa. Geometric means inferred using Bayesian methods and a relaxed molecular clock; ranges in parentheses are 95 % highest posterior density intervals

^b^SFV *pol* and cytB TMRCAs from previous study by our group [26] for comparison

^c^Dating and fossil estimates from Perelman et al. 2011 [31]

### Evaluation of SFV transmission among captive NWP

To investigate potential SFV transmission routes in captive NWP we examined the demographic and epidemiological characteristics of animals housed in 36 different NWP vivaria harboring from 2 to 6 animals each, including 14 vivaria at RIOZOO and 22 at CPRJ (Fig. 5). In most vivaria (26/36, 72.2 %), concordance of infection status was observed, including ten vivaria all containing SFV-positive monkeys and 16 vivaria all housing SFV-negative animals. Interestingly, in some vivaria with circulating SFV, we found monkeys positive by both WB and PCR living with monkeys that were only WB positive (Fig. 5, vivaria 19 and 20). In addition, we found monkeys that were both WB and PCR positive housed with monkeys testing positive by only PCR (Fig. 5, vivaria 16 and 21). Correlations between these discordant test results and animal age, gender, or being wild or captive born was not observed.

In vivaria housing both SFV-infected and uninfected monkeys, we observed a trend of increased infection in older animals (Table 5). However, a significant correlation for this difference was not found. We further analyzed age differences of SFV-infected and uninfected monkeys in the *Cebus* genus, since they represent the majority (36 %, 51/140) of the total samples in our study population. We found that the mean age of SFV-infected *Cebus* (defined by WB and/or PCR-positivity) was significantly higher (9.2 ± 4.1) than that of SFV-negative *Cebus* (2.8 ± 2.7) (*p* = 0.008). Six vivaria harbored male–female pairs and their descendants where at least one parent was SFV-positive, allowing an assessment of horizontal transmission of SFV to offspring born in captivity (Fig. 5). Although SFV-infected offspring were identified in five of these vivaria (83.3 %), patterns of horizontal transmission were not significant. Adult offspring born in captivity when at least one parent was SFV-infected occurred in 86 % (6/7) of cases compared to 60 % (6/10) of juvenile offspring (*p* 0.338) (Table 5). Specimens were not available from infants to evaluate vertical transmission in our study. Our analysis of the effects of being born in captivity *versus* wild-born on infection status did not find any significant differences, which may be confounded by testing of wild-born adult animals and not knowing if infection occurred prior to captivity (Table 5).

**Table 5:** Epidemiological characteristics of monkeys in vivaria with circulating SFV

Characteristic	N	Infected (%)*	*p* value**
Gender			0.999
Male	28	23 (82 %)	
Female	39	32 (82 %)	
*Sexual maturation*			0.187
Adult	45	39 (87 %)	
Juvenile	22	16 (73 %)	
Sexual maturation of offspring***			0.338
Adult	7	6 (86 %)	
Juvenile	10	6 (60 %)	
*Birth origin*			0.168
Captivity	28	21 (75 %)	
Wild	39	34 (87 %)	

* Animals SFV-positive using Western blot and/or PCR testing for any fragment (LTR-gag, polymerase)

** Fisher’s exact test

*** Born in captivity

Four wild-born animals not in vivaria, two adult and two infants, were tested while quarantined. One adult *C. olivaceus* was WB-negative and diagnostic PCR-positive only and one infant *Saimiri* species (2.8-years-old) was negative in all tests. One adult *A. belzebul* and one *Chiropotes* infant (0.4-years-old) were both WB- and *pol* PCR-positive using both *pol* assays. SFV infection of the *Chiropotes* infant suggests a possible mother-to-child transmission but would require additional testing of the mother prior to childbirth for confirmation but those samples were not available.

## Discussion

While NWPs comprise genetically diverse and geographically dispersed species in Central and South America, little is known about the prevalence, geographical distribution, transmission, and evolutionary history of SFV in neotropical monkeys. In our first study [26], we tested a large collection of genomic DNA samples comprising 332 NWP in Brazil belonging to 14 genera of which 24.1 % were found to be SFV-infected using only a screening PCR assay. In that report, we speculated that the lower SFV prevalence in NWP compared to the 70–100 % prevalence reported for OWP may be explained by the higher genetic divergence between SFV infecting different NWP families, about 41 % in *pol* compared to <30 % in Old World monkeys and apes, which could cause more false-negative results when using only the generic screening PCR. The lack of available sequences from many NWP SFV strains for primer design could also explain a lower PCR sensitivity and prevalence. Finally, the capture of NWPs as infants for members in zoo collections or for other reasons could also explain the lower SFV prevalence in that study as little evidence exists for vertical transmission of SFV [17, 35, 36].

Thus, in the current study we also employed serological testing to overcome these potential limitations of screening for evidence of SFV infection using only molecular assays. Our present study found a higher SFV prevalence when determined by serological testing (42 %) than by PCR (35 %), further supporting the viral diversity hypothesis. When testing was done using both serology and PCR we obtained a combined NWP SFV prevalence of 55.8 %, which is closer to that reported for OWP SFV. Despite the better sensitivity observed with the WB assay, which detects antibodies and is thus usually a more generic method for detecting microbial infection, both assays were necessary to more accurately determine SFV infection in NWPs. For example, we observed infected monkeys with discordant WB and PCR results, including those sharing the same vivarium.

Although we did obtain discordant PCR and serological results for some animals, these assays were sensitive enough to detect SFV in all three NWP families. WB-positive and PCR-positive monkeys were identified in both the Atelidae (*Alouatta*) and Pitheciidae (*Cacajao* and *Chiropotes*) families. In contrast, only three of eight Cebidae genera were positive using both tests. The presence of only WB-positivity in some cases could be from low PBMC proviral loads causing false-negative PCR results which are typical in SFV infection, compared to higher levels in other simian retrovirus infections, like SIV and STLV [26, 37]. For example, Stenbak et al. have recently found that SFV proviral loads in PBMC of squirrel monkeys (*Saimiri*) are relatively low but their results are limited by the testing of small numbers of captive animals from a single species. Some animals in our study were only positive in the diagnostic PCR test which amplifies a smaller gene region and may thus be more sensitive for detecting SFV sequences. In addition, because of the small size of NWPs less blood and thus less gDNA was available for input in the molecular tests which may have affected our results. Another possibility is that these animals were exposed, mounted an immune response but were not persistently infected. Genetic heterogeneity of NWP SFV sequences at the primer locations [19] could also explain the false-negative PCR results. Maternal antibodies passed from mother to child may also explain such results and can persist for 6–12 months. However, the majority of these WB-positive only animals were mature with ages ranging from 1.6 to 15.5 years, which does not support this latter possibility. Interestingly, this pattern was observed in 11 different species of which the majority were *Cebus* monkeys. Thus, additional studies are needed to fully understand these discordant results and to measure proviral loads in SFV-infected NWPs.

Interestingly, we also found some monkeys of 10 different species that only tested PCR-positive. For those cases, recent infection could explain the SFV antibody false-negative results with these animals being in the pre-seroconversion phase of infection. For example, in calves and sheep, a recent serological survey showed that all FV-inoculated animals developed Gag-specific antibodies only after 4 weeks post-infection [38]. These results would be expected in younger animals; however, a correlation between age and PCR status was not observed in our study and only one of these discordant animals was less than 2-years-old. Alternatively, the negative serology results may indicate latent infection with SFV in which antibodies are no longer produced. Although such latency is atypical of FV infection and most exogenous retroviral infections, it can be common in type D retroviral infection of macaques with animals becoming seronegative but remaining PCR-positive [39]. Our findings are thus important and suggest that utilization of both serological and PCR testing are currently required for accurate determination of SFV infection in NWPs.

In the present survey, we also identified and characterized novel SFV strains infecting tamarins (*Leontopithecus*), bearded saki monkeys (*Chiropotes* sp.) and uakaris (*Cacajao melanocephalus*), thus enabling the taxonomical classification of SFVcme (from *Cacajao*), SFVlsp (from *Leontopithecus*), and SFVcsp (from *Chiropotes*). Our finding of new, highly divergent SFV in squirrel monkeys that cluster independent of other Cebidae and basal to platyrrhine SFV expands further the viral diversity in neotropical primates and confirms the non-conforming co-evolutionary history of SFV in *Saimiri* [9, 29]. It is not exactly clear why squirrel monkey SFV do not cluster with other Cebidae as expected if they have co-evolved, but which could be due to a possible ancient host switching event from an extinct NWP harboring this variant during the basal Platyrrhini radiation. Our dating estimates of about 41 MYA for the crown Platyrrhini divergence occurring during the Eocene epoch is earlier than that reported for NWP host sequences which were inferred to have diverged ~25 MYA during the Late Oligocene epoch [31]. The earliest primate fossils found in South America anatomically resembled cebids and were estimated to have been present in Bolivia about 26 MYA, which is the minimum boundary for this divergence time estimate [40]. Moreover, the recent finding of NWP molar fossils in Peru morphologically and phylogenetically similar to the Eocene African anthropoid *Talahpithecus* extends the origin of Platyrrhini to the late Eocene ~38–39 MYA [41], which is more consistent with our estimates of ~41 MYA. Combined, these findings support further our ancient host-switching hypothesis and suggest an earlier evolution of NWPs than previously reported using host sequences. More research is needed to explore further the long independent evolution of SFV in this species and NWPs, including expanded sampling of other *Saimiri* species and extant Cebidae.

Phylogenetic analysis also revealed additional evidence of cross-species transmission of SFV among NWPs, considered rare events in OWMAs. One *Leontopithecus* SFV clustered within the *Cebus* SFV radiation and the second *Leontopithecus* SFV clustered with the *Cacajao* (uakaris) in the Pitheciidae clade, instead of with marmoset SFVs as would be expected under a co-evolutionary hypothesis. *Leontopithecus chrysomelas* occasionally associate with *Callithrix kuhli*, *Cebus xanthosternos*, and *Callicebus melanochir* and have a very small range in Bahia and Minas Gerais states, Brazil (http://www.pin.primate.wisc.edu/factsheets/entry/golden-headed_lion_tamarin). Thus, SFV infection of *Leontopithecus* likely represents two independent cross-species transmission events from a capuchin and Pitheciidae species, possibly *Callicebus*, during captivity. Analysis of SFV sequences from wild *Leontopithecus* are required to identify and resolve the evolutionary history of SFV in this species. LTR-*gag* sequences were not obtained for these two monkeys for comparison with the *pol* phylogenies and to evaluate any possible genomic recombination effects in these different genomic regions. The clustering of *Leontopithecus* SFVs with viruses of other genera of the Cebidae family or even with Pitheciidae, together with our previous report of SFVs from *Cebus* grouping with viruses of the Atelidae family [25], further suggest a large plasticity of neotropical primate SFVs to jump between species of disparate Platyrrhini genera or families, compared to that reported for OWMAs.

In our study we also assessed the impact of various demographic factors on SFV transmission and prevalence at two institutions, including gender, sexual maturity, and in wild *versus* captive prior to being housed in vivaria. In vivaria with SFV-infected animals the prevalence ranged from 33 to 100 %. In all cases, the observed differences in the demographic variables were not significant, likely due to small numbers of animals studied at either institution. However, for the largest numbers of monkeys in our study population, capuchins, SFV-infected monkeys were significantly older than uninfected animals. These results are consistent with previous studies in wild chimpanzees and cynomolgus macaques showing an increasing rate of SFV infection with age [17, 36]. The presence of SFV in sexually immature monkeys in our study demonstrated that sexual activity is not the major form of viral transmission and is likely communicated during aggressive behavior as others have proposed for OWMA [35, 36, 42]. Interestingly, we did observe a higher proportion of SFV-positivity using any assay in immature monkeys compared to mature animals suggestive of vertical transmission, but this result was not statistically significant. Nonetheless, Blasse et al. reported mother-to-offspring SFV transmission in wild chimpanzees [43], suggesting this infection route occurs but is likely less efficient. Due to ethical and animal welfare reasons, we were unable to collect specimens from the majority of infants to assess possible mother-to-child transmission. However, one wild *Chiropotes* infant tested both WB- and PCR-positive during routine clinical testing of quarantined animals, suggesting a possible mother-to-child transmission which has not been reported previously for NWMs.

## Conclusions

Using very sensitive serological and molecular tools we expanded significantly the distribution of SFV in NWP in Brazil, including identification of infection in *Leontopithecus, Chiropotes* and *Cacajao.* We also demonstrated by comparison of serological and molecular methods that both assays are currently needed to accurately identify infection with NWP SFV. The addition of more SFV antigens and sequences from other genera may help to improve the diagnostic sensitivities of each method. These new tools used in our study will also facilitate investigation of zoonotic infection of persons working with or hunting NWPs. By reconstructing the evolutionary histories we presented phylogenetic evidence that like SFV from OWMAs, SFV in NWP have an ancient codivergence with rare host switching likely occurring at the radiation of NWPs for squirrel monkeys. Sequences from additional NWPs are required to further resolve the natural history of SFV in NWPs, especially the long, independent evolution of SFV in *Saimiri*. While we showed that SFV infection increased with age in *Cebus* monkeys, probably during aggressive behaviors, more work is also required to better understand transmission routes in NWPs.

## References

[1] Linial ML (1999). Foamy viruses are unconventional retroviruses. J Virol.

[2] Khan AS (2009). Simian foamy virus infection in humans: prevalence and management. Expert Rev Anti Infect Ther.

[3] Switzer WM, Heneine W, Liu D (2011). Foamy virus infection of humans. Molecular detection of human viral pathogens.

[4] Jones-Engel L, May CC, Engel GA, Steinkraus KA, Schillaci MA, Fuentes A, Rompis A, Chalise MK, Aggimarangsee N, Feeroz MM (2008). Diverse contexts of zoonotic transmission of simian foamy viruses in Asia. Emerg Infect Dis.

[5] Wu Z, Ren X, Yang L, Hu Y, Yang J, He G, Zhang J, Dong J, Sun L, Du J (2012). Virome analysis for identification of novel mammalian viruses in bat species from Chinese provinces. J Virol.

[6] Katzourakis A, Gifford RJ, Tristem M, Gilbert MT, Pybus OG (2009). Macroevolution of complex retroviruses. Science.

[7] Han GZ, Worobey M (2012). An endogenous foamy virus in the aye–aye (Daubentonia madagascariensis). J Virol.

[8] Han GZ, Worobey M (2014). Endogenous viral sequences from the cape golden mole (Chrysochloris asiatica) reveal the presence of foamy viruses in all major placental mammal clades. PLoS One.

[9] Katzourakis A, Aiewsakun P, Jia H, Wolfe ND, LeBreton M, Yoder AD, Switzer WM (2014). Discovery of prosimian and afrotherian foamy viruses and potential cross species transmissions amidst stable and ancient mammalian co-evolution. Retrovirology.

[10] Han GZ, Worobey M (2012). An endogenous foamy-like viral element in the coelacanth genome. PLoS Pathog.

[11] Schartl M, Walter RB, Shen Y, Garcia T, Catchen J, Amores A, Braasch I, Chalopin D, Volff JN, Lesch KP (2013). The genome of the platyfish, Xiphophorus maculatus, provides insights into evolutionary adaptation and several complex traits. Nat Genet.

[12] Hooks JJ, Gibbs CJ (1975). The foamy viruses. Bacteriol Rev.

[13] Kupiec JJ, Kay A, Hayat M, Ravier R, Peries J, Galibert F (1991). Sequence analysis of the simian foamy virus type 1 genome. Gene.

[14] McClure MO, Bieniasz PD, Schulz TF, Chrystie IL, Simpson G, Aguzzi A, Hoad JG, Cunningham A, Kirkwood J, Weiss RA (1994). Isolation of a new foamy retrovirus from orangutans. J Virol.

[15] Broussard SR, Comuzzie AG, Leighton KL, Leland MM, Whitehead EM, Allan JS (1997). Characterization of new simian foamy viruses from African nonhuman primates. Virology.

[16] Jones-Engel L, Engel GA, Heidrich J, Chalise M, Poudel N, Viscidi R, Barry PA, Allan JS, Grant R, Kyes R (2006). Temple monkeys and health implications of commensalism, Kathmandu, Nepal. Emerg Infect Dis.

[17] Liu W, Worobey M, Li Y, Keele BF, Bibollet-Ruche F, Guo Y, Goepfert PA, Santiago ML, Ndjango JB, Neel C (2008). Molecular ecology and natural history of simian foamy virus infection in wild-living chimpanzees. PLoS Pathog.

[18] Leendertz SA, Junglen S, Hedemann C, Goffe A, Calvignac S, Boesch C, Leendertz FH (2010). High prevalence, coinfection rate, and genetic diversity of retroviruses in wild red colobus monkeys (Piliocolobus badius badius) in Tai National Park, Cote d’Ivoire. J Virol.

[19] Moreira MA, Bonvicino CR, Soares MA, Seuanez HN (2010). Genetic diversity of neotropical primates: phylogeny, population genetics, and animal models for infectious diseases. Cytogenet Genome Res.

[20] Hooks JJ, Gibbs CJ, Chou S, Howk R, Lewis M, Gajdusek DC (1973). Isolation of a new simian foamy virus from a spider monkey brain culture. Infect Immun.

[21] Barahona H, Garcia FG, Melendez LV, King NW, Ingalls JK (1976). Isolation and characterization of lymphocyte associated foamy virus from a red uakari monkey (Cacajao rubicundus). J Med Primatol.

[22] Marczynska B, Jones CJ, Wolfe LG (1981). Syncytium-forming virus of common marmosets (Callithrix jacchus jacchus). Infect Immun.

[23] Thumer L, Rethwilm A, Holmes EC, Bodem J (2007). The complete nucleotide sequence of a New World simian foamy virus. Virology.

[24] Pacheco B, Finzi A, McGee-Estrada K, Sodroski J (2010). Species-specific inhibition of foamy viruses from South American monkeys by New World Monkey TRIM5{alpha} proteins. J Virol.

[25] Switzer WM, Salemi M, Shanmugam V, Gao F, Cong ME, Kuiken C, Bhullar V, Beer BE, Vallet D, Gautier-Hion A (2005). Ancient co-speciation of simian foamy viruses and primates. Nature.

[26] Muniz CP, Troncoso LL, Moreira MA, Soares EA, Pissinatti A, Bonvicino CR, Seuanez HN, Sharma B, Jia H, Shankar A (2013). Identification and characterization of highly divergent simian foamy viruses in a wide range of new world primates from Brazil. PLoS One.

[27] Stenbak CR, Craig KL, Ivanov SB, Wang X, Soliven KC, Jackson DL, Gutierrez GA, Engel G, Jones-Engel L, Linial ML (2014). New World simian foamy virus infections in vivo and in vitro. J Virol.

[28] Hussain AI, Shanmugam V, Bhullar VB, Beer BE, Vallet D, Gautier-Hion A, Wolfe ND, Karesh WB, Kilbourn AM, Tooze Z (2003). Screening for simian foamy virus infection by using a combined antigen Western blot assay: evidence for a wide distribution among Old World primates and identification of four new divergent viruses. Virology.

[29] Ghersi BM, Jia H, Aiewsakun P, Katzourakis A, Mendoza P, Busch DG, Kasper MR, Switzer WM. Wide distribution and ancient evolutionary history of simian foamy viruses in New World primates. Retrovirology. In press. 10.1186/s12977-015-0214-0 PMC4627628 26514626

[30] Tamura K, Stecher G, Peterson D, Filipski A, Kumar S (2013). MEGA6: molecular evolutionary genetics analysis version 6.0. Mol Biol Evol.

[31] Perelman P, Johnson WE, Roos C, Seuanez HN, Horvath JE, Moreira MA, Kessing B, Pontius J, Roelke M, Rumpler Y (2011). A molecular phylogeny of living primates. PLoS Genet.

[32] Auricchio P. Primates of Brazil. São Paulo: Terra Brasilis Educational material of Commerce and Editora Ltda—ME; 1995. pp. 168. ISBN 85-85712-01-5.

[33] Alfaro JW, Silva JD, Rylands AB (2012). How different are robust and gracile capuchin monkeys? An argument for the use of sapajus and cebus. Am J Primatol.

[34] Finstermeier K, Zinner D, Brameier M, Meyer M, Kreuz E, Hofreiter M, Roos C (2013). A mitogenomic phylogeny of living primates. PLoS One.

[35] Murray SM, Linial ML (2006). Foamy virus infection in primates. J Med Primatol.

[36] Hood S, Mitchell JL, Sethi M, Almond NM, Cutler KL, Rose NJ (2013). Horizontal acquisition and a broad biodistribution typify simian foamy virus infection in a cohort of Macaca fascicularis. Virol J.

[37] Rua R, Betsem E, Gessain A (2013). Viral latency in blood and saliva of simian foamy virus-infected humans. PLoS One.

[38] Materniak M, Hechler T, Lochelt M, Kuzmak J (2013). Similar patterns of infection with bovine foamy virus in experimentally inoculated calves and sheep. J Virol.

[39] Lerche NW, Yee JL, Jennings MB (1994). Establishing specific retrovirus-free breeding colonies of macaques: an approach to primary screening and surveillance. Lab Anim Sci.

[40] Perez SI, Tejedor MF, Novo NM, Aristide L (2013). Divergence times and the evolutionary radiation of new world monkeys (Platyrrhini, Primates): an analysis of fossil and molecular data. PLoS One.

[41] Bond M, Tejedor MF, Campbell KE, Chornogubsky L, Novo N, Goin F (2015). Eocene primates of South America and the African origins of New World monkeys. Nature..

[42] Calattini S, Wanert F, Thierry B, Schmitt C, Bassot S, Saib A, Herrenschmidt N, Gessain A (2006). Modes of transmission and genetic diversity of foamy viruses in a Macaca tonkeana colony. Retrovirology.

[43] Blasse A, Calvignac-Spencer S, Merkel K, Goffe AS, Boesch C, Mundry R, Leendertz FH (2013). Mother-offspring transmission and age-dependent accumulation of simian foamy virus in wild chimpanzees. J Virol.

